# Therapeutic Drug Monitoring of Olanzapine: Effects of Clinical Factors on Plasma Concentrations in Psychiatric Patients

**DOI:** 10.1097/FTD.0000000000001227

**Published:** 2024-06-03

**Authors:** Nicolas Ansermot, Harish Vathanarasa, Setareh Ranjbar, Mehdi Gholam, Séverine Crettol, Frederik Vandenberghe, Franziska Gamma, Kerstin Jessica Plessen, Armin von Gunten, Philippe Conus, Chin B. Eap

**Affiliations:** * Unit of Pharmacogenetics and Clinical Psychopharmacology, Centre for Psychiatric Neuroscience, Department of Psychiatry, Lausanne University Hospital, University of Lausanne, Prilly, Switzerland;; † Psychiatric Epidemiology and Psychopathology Research Centre, Department of Psychiatry, Lausanne University Hospital, University of Lausanne, Prilly, Switzerland;; ‡ Les Toises Psychiatry and Psychotherapy Centre, Lausanne, Switzerland;; § Service of Child and Adolescent Psychiatry, Department of Psychiatry, Lausanne University Hospital, University of Lausanne, Prilly, Switzerland;; ‖ Service of Old Age Psychiatry, Department of Psychiatry, Lausanne University Hospital, University of Lausanne, Prilly, Switzerland;; ¶ Service of General Psychiatry, Department of Psychiatry, Lausanne University Hospital, University of Lausanne, Prilly, Switzerland;; ** Centre for Research and Innovation in Clinical Pharmaceutical Sciences, University of Lausanne, Lausanne, Switzerland;; †† School of Pharmaceutical Sciences, University of Geneva, Geneva, Switzerland; and; ‡‡ Institute of Pharmaceutical Sciences of Western Switzerland, University of Geneva, University of Lausanne, Geneva and Lausanne, Switzerland.

**Keywords:** olanzapine, plasma concentrations, clinical factors, therapeutic drug monitoring

## Abstract

**Background:**

Therapeutic drug monitoring (TDM) is strongly recommended for olanzapine due to its high pharmacokinetic variability. This study aimed to investigate the impact of various clinical factors on olanzapine plasma concentrations in patients with psychiatric disorders.

**Methods:**

The study used TDM data from the PsyMetab cohort, including 547 daily dose–normalized, steady-state, olanzapine plasma concentrations (C:D ratios) from 248 patients. Both intrinsic factors (eg, sex, age, body weight) and extrinsic factors (eg, smoking status, comedications, hospitalization) were examined. Univariate and multivariable, linear, mixed-effects models were employed, with a stepwise selection procedure based on Akaike information criterion to identify the relevant covariates.

**Results:**

In the multivariable model (based on 440 observations with a complete data set), several significant findings emerged. Olanzapine C:D ratios were significantly lower in smokers (β = −0.65, *P* < 0.001), valproate users (β = −0.53, *P* = 0.002), and inpatients (β = −0.20, *P* = 0.025). Furthermore, the C:D ratios decreased significantly as the time since the last dose increased (β = −0.040, *P* < 0.001). The male sex had a significant main effect on olanzapine C:D ratios (β = −2.80, *P* < 0.001), with significant interactions with age (β = 0.025, *P* < 0.001) and body weight (β = 0.017, *P* = 0.011). The selected covariates explained 30.3% of the variation in C:D ratios, with smoking status accounting for 7.7% and sex contributing 6.9%. The overall variation explained by both the fixed and random parts of the model was 67.4%. The model facilitated the prediction of olanzapine C:D ratios based on sex, age, and body weight.

**Conclusions:**

The clinical factors examined in this study, including sex, age, body weight, smoking status, and valproate comedication, remarkably influence olanzapine C:D ratios. Considering these factors, in addition to TDM and the clinical situation, could be important for dose adjustment.

## Abstract 2

Supplemental Digital Content is Available in the Text.

## INTRODUCTION

Olanzapine is a commonly used atypical antipsychotic medication for the treatment of schizophrenia and manic episodes in bipolar I disorder. The recommended oral daily doses range from 5 to 20 mg.^1^ It has been found to be effective in treating both positive and negative symptoms of schizophrenia and has a lower risk of extrapyramidal symptoms and hyperprolactinemia compared with other antipsychotics.^2^ However, olanzapine is associated with significant weight gain, metabolic disorders, sedation, anticholinergic effects, and cardiovascular effects.^2,3^

Olanzapine exhibits high pharmacokinetic variability, which can be attributed to clinical and genetic factors.^4–6^ Its mean oral bioavailability is approximately 60%,^5^ and it takes 5–8 hours to reach maximum plasma concentration. The mean ± SD apparent volume of distribution is 1148 ± 360 L, and the mean ± SD elimination half-life is 33 ± 10 hours.^4^ Olanzapine is primarily metabolized in the liver, mainly by cytochrome P450 (CYP) 1A2 and uridine diphosphate glucuronosyltransferase (UGT) 1A4, with contributions from other enzymes, including CYP2C8, CYP2D6, CYP3A43, UGT2B10, and flavin monooxygenases 1 and 3.^6–8^

Several studies have found associations between olanzapine plasma concentrations and therapeutic response as well as adverse reactions,^9–11^ although not all studies have observed these associations.^12^ The *Arbeitsgemeinschaft für Neuropsychopharmakologie und Pharmakopsychiatrie* consensus guidelines strongly recommend therapeutic drug monitoring (TDM) of olanzapine plasma levels for dose adjustment, particularly in cases of adverse drug reactions, nonresponse to treatment, or drug–drug interactions. The recommended therapeutic reference range is 20–80 ng/mL.^13^ However, a recent systematic review suggested a narrower therapeutic reference range of 20–40 ng/mL for optimal treatment response in patients with schizophrenia and schizophrenia spectrum disorders.^14^ The authors added that higher plasma concentrations are generally well tolerated and may not require dose reduction if the response and tolerance are satisfactory.

Several studies have investigated the potential influence of clinical factors on olanzapine pharmacokinetics, including sex, age, smoking status, or comedications. However, many of these studies have limitations. Some studies did not consider important factors, such as smoking status,^15^ age,^12^ body weight,^15–19^ or comedications.^20,21^ Other studies did not report steady-state conditions,^17,18^ included a small sample size,^22–24^ excluded smokers,^16^ or quantified olanzapine with a low-specificity analytical method.^10,12,19,25^

The aim of this study was to investigate the effects of intrinsic factors (sex, age, body weight) and extrinsic factors (smoking status, comedications, hospitalization, sampling time after the last dose) on daily dose–normalized, steady-state, olanzapine plasma concentrations (C:D ratios) in psychiatric patients. In addition, the study explored the potential influence of C-reactive protein (CRP), a marker of inflammation that could potentially lead to decreased CYP1A2 activity,^26,27^ in a subgroup of patients.

## MATERIALS AND METHODS

### Study Design and Patients

An ongoing, observational, prospective study called PsyMetab has been conducted since 2007 in the Department of Psychiatry at the Lausanne University Hospital and a private mental health care center in Lausanne, Switzerland (Les Toises). This study uses clinical data and blood samples collected during routine follow-up of patients with psychiatric disorders receiving psychotropic treatment, as previously described.^28^ The study received approval from the Ethics Committee of the Canton de Vaud (2017-01301), and the patients provided written informed consent.

For this study, inpatients and outpatients from this cohort who were prescribed oral olanzapine and had at least 1 olanzapine plasma concentration measurement during routine TDM between 2008 and 2019 were included, without any age restrictions. Exclusion criteria were as follows: unknown olanzapine daily dose, plasma concentration not at steady state (last dose change less than 5 days before blood sampling) or unknown time of the last dose change, olanzapine administered intramuscularly less than 5 days before blood sampling, sampling time after the last dose <9 or >30 hours or unreliable information about timing, and olanzapine plasma concentration below the limit of quantification (0.5 ng/mL), which could indicate nonadherence to treatment (see **Figure S1, Supplemental Digital Content 1**, http://links.lww.com/TDM/A770, for the flowchart depicting the inclusion of olanzapine plasma concentrations). It is worth noting that no patients in the study received long-acting injectable olanzapine, as this formulation is not available in Switzerland.

Clinical information, including intrinsic factors (sex, age, body weight) and extrinsic factors (smoking status, comedications, hospitalization, sampling time after the last dose), was gathered from the patients' medical records. Comedications that might potentially influence olanzapine plasma concentrations were identified, such as levomepromazine, fluoxetine, and paroxetine (strong CYP2D6 inhibitors), esomeprazole (weak CYP1A2 inducer), and valproate (interaction mechanism unclear).^29–31^ No patient took strong CYP1A2 inhibitors, such as fluvoxamine. Patients who were taking exogenous estrogens (CYP1A2 inhibitors), such as oral contraceptive pills, did not have information available in the medical records.^32^ For patients with multiple olanzapine plasma measurements, the comedications taken at the time of each measurement were included in the analyses.

### Quantification of Olanzapine and CRP Plasma Concentrations

The blood samples were collected in tubes containing ethylenediaminetetraacetic acid. After centrifugation, plasma samples were stored at −20°C until analysis, which was performed for olanzapine during routine TDM (ie, 1–4 days after blood sampling). Olanzapine plasma concentrations were quantified by ultra-high-performance liquid chromatography (UHPLC) (Waters ACQUITY UPLC system) coupled with tandem mass spectrometry (MS/MS) (Waters TQD or Waters Xevo TQ-S). The method was fully validated according to international guidelines with a limit of quantification of 0.5 ng/mL.^33^ High-sensitivity CRP was quantified retrospectively by immunoassay using an Indiko plus Thermo Scientific analyzer (Thermo Fisher Scientific, Reinach, Switzerland) using available plasma samples stored at −20°C.

### Statistical Analyses

The outcome of interest was olanzapine C:D ratio, expressed in (ng/mL)/mg, which is considered, in adherent patients, as an index of the capacity of the body to eliminate the drug, with lower and higher values associated with increased or reduced elimination, respectively. Because of the longitudinal design of the study, half of the patients underwent repeated observations. All observations were included in the analyses using linear mixed-effects models fitted by the maximum likelihood method to detect the effects of different covariates on the olanzapine C:D ratios. In these models, personal ID was used as a random intercept to account for the patients' intraclass correlations. The analyses were first performed using univariate models, and then, a multivariable model was built. Subgroup analyses were performed when the interaction between sex and smoking status was significant. To select the most relevant and informative covariates in the multivariable model, a stepwise model selection procedure (backward selection) based on Akaike information criterion was used. A *P* value of < 0.05 was considered statistically significant. Marginal and conditional R^2^ values were reported as measures of goodness of fit for the multivariable model. The former represents the percentage of variation in the outcome explained by the fixed parts of the model (covariates included in the model), and the latter shows the total percentage of variation explained by the fixed and random parts of the model (covariates included in the model and the random intercept to account for the clustering structure in the data). The interaction plots for the full model are also illustrated. Statistical analyses were performed using the R language and environment for statistical computing 4.1.2.^34^ Linear mixed-effects models were fitted using the *lme* function of the nlme package,^35^ and variable selection was performed using the *stepAIC* function of the MASS package.^36^

## RESULTS

### Study Population

A total of 547 olanzapine plasma samples (248 patients) were included in the analyses. The proportions of plasma concentrations measured in male patients and in smokers were 54% and 59%, respectively (Table 1). The mean age of the patients was 42 years (range 13–90 years), and the mean body weight was 73 kg (range 36–131 kg), including repeated measures. The most prescribed comedication that could influence olanzapine plasma concentrations was valproate, which was present in 10.7% of the observations. The mean olanzapine daily dose and plasma concentration were 16.3 mg (range 1.25–40 mg) and 34.0 ng/mL (range 1–138 ng/mL), respectively. The proportion of plasma concentrations within the *Arbeitsgemeinschaft für Neuropsychopharmakologie und Pharmakopsychiatrie* therapeutic reference range (20–80 ng/mL)^13^ was 64%, whereas 31% were below and 5% were above this range (see **Figure S2, Supplemental Digital Content 1**, http://links.lww.com/TDM/A770, which shows olanzapine plasma concentrations as a function of the daily dose). When the new therapeutic reference range of 20–40 ng/mL was considered,^14^ 32% of the concentrations were above the recommended range, and only 37% were within. Olanzapine C:D ratios varied up to 74 fold [0.1–7.4 (ng/mL)/mg], with a mean value of 2.2 (ng/mL)/mg. In a subgroup of 116 patients (156 observations), median (interquartile range) CRP was 1.95 (0.87–4.19) mg/L, with a range of 0.06–50.3 mg/L.

**TABLE 1.** Characteristics of the Entire Sample Based on the Number of Observations (n = 547)

Characteristics	Values
Males	297 (54.3)
Age (y)	42 (16), 13–90
Weight (kg) (NA = 12)	73 (16), 36–131
Olanzapine daily dose (mg)	16.3 (7.9), 1.25–40
Olanzapine plasma concentration (ng/mL)	34.0 (23.0), 1–138
Olanzapine plasma concentration/daily dose (C:D) [(ng/mL)/mg]	2.2 (1.1), 0.1–7.4
Sampling time after last dose (h)	14.3 (3.8), 9.0–27.5
Inpatients	301 (55.0)
Smokers (NA = 35)	300 (58.6)
Valproate (NA = 72)	51 (10.7)
Esomeprazole (NA = 72)	37 (7.8)
Levomepromazine (NA = 72)	14 (2.9)
Fluoxetine (NA = 72)	8 (1.7)
Paroxetine (NA = 72)	9 (1.9)
At least 1 CYP2D6 inhibitor (NA = 72)*	30 (6.3)

The numbers of patients with 1, 2, 3, 4, and 5–10 observations were 123, 59, 21, 21, and 24, respectively. Data are presented as counts (percentages) for categorical variables and as means (SDs) with ranges for continuous variables.

* CYP2D6 inhibitors: levomepromazine, fluoxetine, and paroxetine.

CYP, cytochrome P450; NA, not available.

### Effects of Clinical Factors on Olanzapine C:D Ratios

The results of the univariate analyses are presented in Table 2 and **Supplemental Digital Content 1** (see **Figures S3, S4, S5 and S6**, http://links.lww.com/TDM/A770). Mean olanzapine C:D ratios were significantly lower in smokers (−33%), in male patients (−21%), and in inpatients (−14%). Olanzapine C:D ratios increased significantly with age, but body weight had no effect. A trend toward lower mean olanzapine C:D ratios was observed in patients taking valproate (−16%), but no influence was observed for esomeprazole and CYP2D6 inhibitors. As expected, the olanzapine C:D ratio decreased significantly with the sampling time after the last dose.

**TABLE 2.** The Effects of Clinical Factors on Daily Dose-Normalized Olanzapine Plasma Concentrations (C:D Ratios) Using Univariate Analyses (547 Observations and 248 Patients)

Predictors	Estimates (β)*	CI 95%	*P*
Males	**−0.52**	**−0.77 to -0.26**	**<0.001**
Intercept	2.47	2.28 to 2.66	<0.001
Smokers (NA = 35)	**−0.87**	**−1.07 to -0.67**	**<0.001**
Intercept	2.66	2.49 to 2.82	<0.001
Inpatients	**−0.32**	**−0.49 to -0.16**	**<0.001**
Intercept	2.37	2.21 to 2.53	<0.001
Age (y)	**0.013**	**0.006** to **0.021**	**<0.001**
Intercept	1.63	1.29 to 1.97	<0.001
Sampling time after last dose (h)	**−0.044**	**−0.065 to -0.024**	**<0.001**
Intercept	2.81	2.50 to 3.13	<0.001
Weight (kg) (NA = 12)	0.001	−0.006 to 0.008	0.78
Intercept	2.11	1.56 to 2.67	<0.001
Valproate (NA = 72)	−0.34	−0.68 to 0.01	0.054
Intercept	2.18	2.05 to 2.32	<0.001
Esomeprazole (NA = 72)	−0.16	−0.54 to 0.22	0.42
Intercept	2.16	2.02 to 2.30	<0.001
At least 1 CYP2D6 inhibitor (NA = 72)†	−0.14	−0.54 to 0.26	0.50
Intercept	2.16	2.02 to 2.29	<0.001

For binary covariates, the relative difference of mean olanzapine C:D ratios between 2 categories (eg, males versus females) was calculated by dividing the β value (estimate of the alternative group) by the intercept (reference group) and expressed as a percentage (eg, men had C:D ratios −0.52/2.47 = −21% lower than women). See Results section for the relative differences obtained with the other binary covariates.

Statistically significant covariates (except intercepts) are in bold (*P* < 0.05).

* Effect of the predictor on olanzapine C:D ratios expressed in (ng/mL)/mg if the predictor is present (for binary covariates) or for each unit increment of the predictor (for continuous covariates). For example, a man will have an olanzapine C:D ratio of −0.52 (ng/mL)/mg lower than a woman in the univariate model.

† CYP2D6 inhibitors: levomepromazine, fluoxetine, paroxetine.

CI: confidence interval; CYP: cytochrome P450; NA: not available.

**FIGURE 1.**
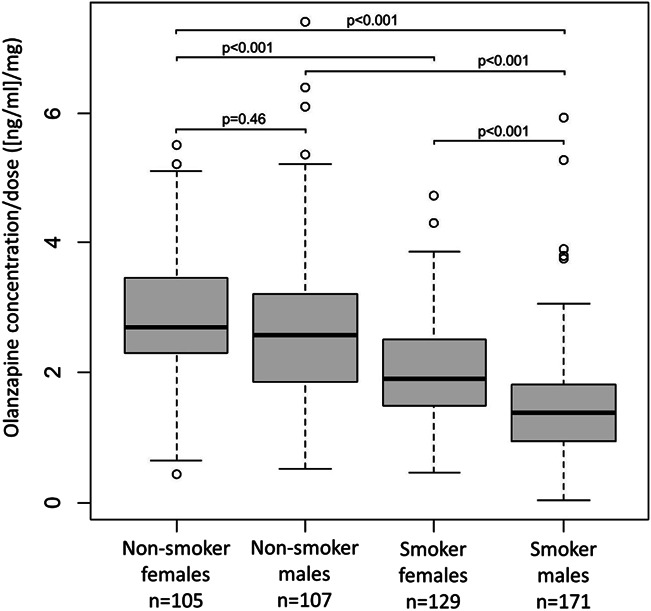
Combined effects of sex and smoking status on daily dose–normalized olanzapine plasma concentrations (C:D ratios). The *P*-values are the results of the fitted linear mixed-effects models.

Subgroup analyses were performed because of the significant interaction between sex and smoking status. When the effects of sex and smoking status were combined and the most distinct subgroups were compared, male smokers had mean olanzapine C:D ratios significantly lower (−45%) than female nonsmokers (Fig. 1). Descriptive statistics of the daily doses, plasma concentrations, and C:D ratios in subgroups of patients according to sex and smoking status are presented in **Supplemental Digital Content 1** (see **Table S1**, http://links.lww.com/TDM/A770). In subgroup analyses by sex, olanzapine C:D ratios increased significantly with age and body weight in men but not in female patients (see **Figures S4 and S5, Supplemental Digital Content 1**, http://links.lww.com/TDM/A770).

In the multivariable model, 440 olanzapine C:D ratios (212 patients) with complete data sets for all covariates were included (Table 3). Olanzapine C:D ratios were significantly lower in smokers (β = −0.65, *P* < 0.001), in patients taking valproate (β = −0.53, *P* = 0.002) and in inpatients (β = −0.20, *P* = 0.025). The main effect of male sex on olanzapine C:D ratios was significant (β = −2.80, *P* < 0.001), with significant interactions with age (β = 0.025, *P* < 0.001) and body weight (β = 0.017, *P* = 0.011). Olanzapine C:D ratios decreased significantly with sampling time after the last dose (β = −0.040, *P* < 0.001). Other covariates (esomeprazole and at least 1 CYP2D6 inhibitor) and the interaction between sex and smoking status were not selected using the stepwise multivariable model. The variation in the C:D ratios explained by the selected covariates was 30.3%, with smoking status and sex accounting for 7.7% and 6.9%, respectively. The total variation explained by the fixed and random parts of the model was 67.4%. Similar results were obtained when body mass index was included in the multivariable model instead of body weight (see **Table S2, Supplemental Digital Content 1**, http://links.lww.com/TDM/A770).

**TABLE 3.** The Effects of Clinical Factors on Daily Dose-Normalized Olanzapine Plasma Concentrations (C:D Ratios) Using Multivariable Analysis (440 Observations and 212 Patients)

Predictors†	Estimates (β)‡	CI 95%	*P*
**Males**	**−2.80**	**−3.94 to -1.65**	**<0.001**
Age (y)	−0.008	−0.018 to 0.002	0.13
Age*males (y)	**0.025**	**0.012 to 0.038**	**<0.001**
Weight (kg)	−0.005	−0.014 to 0.004	0.29
Weight*males (kg)	**0.017**	**0.004 to 0.031**	**0.011**
Sampling time after last dose (h)	**−0.040**	**−0.061 to -0.019**	**<0.001**
**Inpatients**	**−0.20**	**−0.37 to -0.03**	**0.025**
**Smokers**	**−0.65**	**−0.85 to -0.44**	**<0.001**
**Valproate**	**−0.53**	**−0.85 to -0.20**	**0.002**
Intercept	4.14	3.31 to 4.97	<0.001

Marginal R^2^ (the amount of variation explained by the fixed parts of the model) and conditional R^2^ (the amount of variation explained by both the fixed and random parts) were 0.303 and 0.674, respectively. The intraclass correlation coefficient was 0.53.

Statistically significant covariates (except intercepts) are in bold (*P* < 0.05).

† Other covariates (esomeprazole and at least 1 cytochrome P450 2D6 inhibitor) and the interaction between sex and smoking status were included in the analyses but were not selected by the stepwise process of the model.

‡ Effect of the predictor on olanzapine C:D ratios expressed in (ng/mL)/mg if the predictor was present (for binary covariates) or for each unit increment of the predictor (for continuous covariates). If the interaction is significant, it should also be considered. For example, the effect of male sex on olanzapine C:D ratios depends on the age and body weight of the patient and can be calculated as follows: −2.80 + 0.025*age + 0.017*weight.

CI: confidence interval.

**FIGURE 2.**
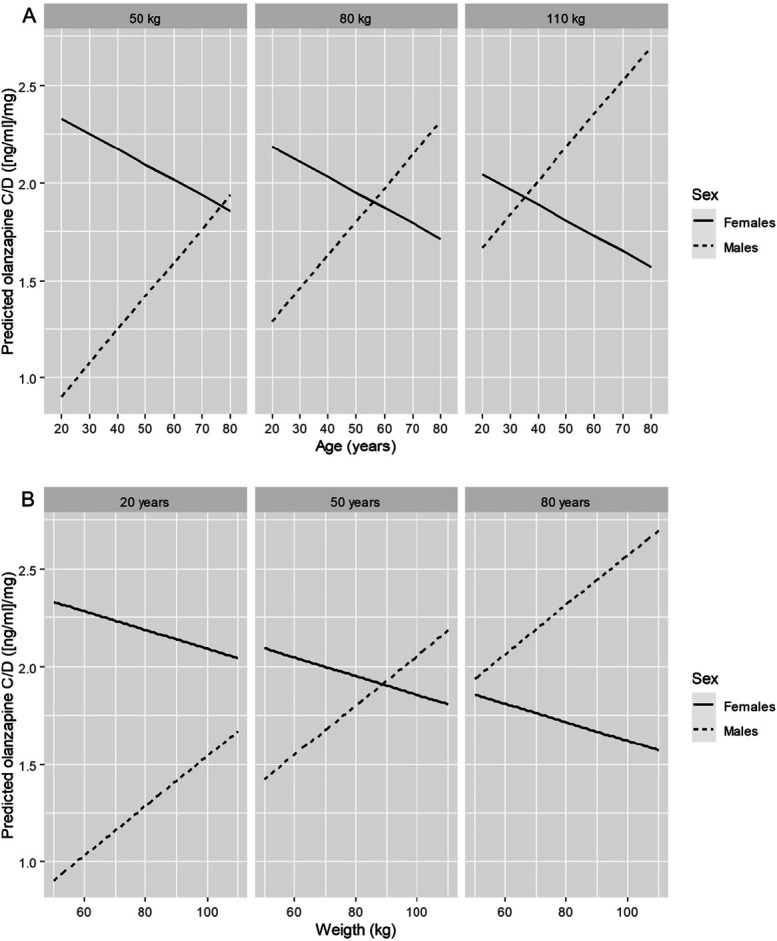
Interaction plots showing the marginal effects of age (A) and body weight (B) on daily dose–normalized olanzapine plasma concentrations (C:D ratios) using separate predicted data for male and female patients of different ages and body weights. The predictions were based on the multivariable analysis presented in Table 3 and adjusted for smokers, inpatients, nonvalproate users, and mean sampling time after the last dose of 14.2 hours.

To better visualize the effects of the interactions between sex, age, and body weight, Figure 2 shows the predicted olanzapine C:D ratios separately for male and female patients of different ages and body weights. Olanzapine C:D ratios increased with age and body weight in male patients and decreased with age and body weight in female patients. For young patients (eg, 20 years), C:D ratios were lower in male patients regardless of body weight (range tested 50–110 kg); for intermediate ages (eg, 50 years), C:D ratios were lower in male patients with low body weight and higher in male patients with high body weight. For elderly patients (eg, 80 years), C:D ratios were higher in male patients regardless of body weight (50–110 kg). An online data visualization application for the olanzapine C:D ratios based on the full multivariable model is provided in **Supplemental Digital Content 2**, https://uppc.shinyapps.io/olanzapine/.

In a subgroup of 116 patients (156 observations), CRP as a continuous variable was not associated with olanzapine C:D ratios in the univariate analysis (see **Figure S7, Supplemental Digital Content 1**, http://links.lww.com/TDM/A770) and was not selected by the stepwise process of the multivariable model (data not shown). However, when CRP was divided into quartiles, the olanzapine C:D ratios were significantly higher in quartiles 3 and 4 than in quartile 1 in the univariate analysis (see **Table S3 and Figure S8, Supplemental Digital Content 1**, http://links.lww.com/TDM/A770), but only quartile 3 was significantly higher than quartile 1 in the multivariable model (see **Table S4, Supplemental Digital Content 1**, http://links.lww.com/TDM/A770).

## DISCUSSION

### Effects of Clinical Factors on Olanzapine C:D Ratios

This study identified important intrinsic and extrinsic factors that influence olanzapine C:D ratios in patients with psychiatric disorders. First, smokers had significantly lower olanzapine C:D ratios than nonsmokers. Our results are consistent with those of prior studies showing that smokers had 23%–55% higher olanzapine clearance,^37,38^ 31%–53% lower olanzapine C:D ratios,^17,18,30^ or lower olanzapine plasma concentrations.^12,20,21^ Cigarette smoke is a potent inducer of CYP1A2 activity,^39^ one of the major enzymes involved in olanzapine metabolism,^40^ due to the binding of polycyclic aromatic hydrocarbons to the aryl hydrocarbon receptor.^41^ Therefore, when individuals quit smoking, it is necessary to decrease the dose of olanzapine, with the help of TDM, to avoid adverse drug reactions associated with elevated olanzapine concentrations.^41^

Sex is another significant factor that influences the olanzapine C:D ratios and interacts with age and weight. Predicted olanzapine C:D ratios were found to be lower in young male patients compared with young female patients but higher in old male patients compared with old female patients. Earlier studies have reported 23%–38% higher olanzapine clearance in male patients,^37,38^ 11%–43% higher olanzapine C:D ratios in female patients,^15–18,30^ or 26% higher olanzapine plasma concentrations in female patients.^21^ However, most of these studies did not consider the interaction between sex, age, and body weight. The higher olanzapine levels predicted in young female patients could be attributed to lower CYP1A2 enzyme activity compared with male patients,^42^ likely due to the inhibition of CYP1A2 by estrogens.^32^ A majority of patients older than 55 years were included in a study that found no significant influence of sex on olanzapine plasma concentrations.^20^ This lack of influence may be attributed to lower estrogen levels in older female patients, which could neutralize the difference between male and female patients. Other sex-specific pharmacokinetic factors, such as the volume of distribution, may also contribute to the differences between sexes. For lipophilic drugs like olanzapine, female patients tend to have a greater volume of distribution due to a higher proportion of adipose tissue. This can result in drug accumulation in adipose tissue, potentially prolonging the half-life.^43,44^

Predicted olanzapine C:D ratios were observed to increase with age in male patients but decrease with age in female patients. Previous studies investigating the effect of age generally reported higher olanzapine C:D ratios^15–18,30^ or olanzapine plasma concentrations^21^ in older patients, although many of them did not consider the interaction with sex. Simulated data from a study indicated that expected olanzapine C:D ratios increased with age in both sexes.^15^ Population pharmacokinetic analyses did not observe a significant effect of age on olanzapine clearance.^37,38^ However, another study found that age had a significant influence on O-desmethyl-olanzapine concentrations but not olanzapine plasma concentrations.^20^ The authors suggested that discrepancies between studies might be attributed to variations in sample sizes and age distributions. Physiological changes associated with aging, such as reduced hepatic blood flow, decreased enzymatic activity, and decreased liver mass,^44^ can contribute to higher drug levels in the elderly,^45^ which could explain the results predicted in male patients in our study. In addition, the volume of distribution increases with age due to a higher proportion of adipose tissue, leading to a longer half-life for lipid-soluble drugs like olanzapine.^46^

Predicted olanzapine C:D ratios were observed to increase with body weight in male patients but decrease with body weight in female patients. However, the influence of body weight on olanzapine plasma levels is inconsistent across previous studies. Some studies have reported a positive correlation between body weight and plasma levels,^21^ whereas others have observed a negative correlation.^20^ In other studies, no significant correlation between body weight and olanzapine plasma levels^30^ or clearance^37,38^ was observed. Several physiological changes are observed in obese patients, which could potentially influence the distribution and clearance of certain drugs.^47^ Clearance of drugs primarily metabolized by UGT, such as olanzapine, appears to be higher in obese patients, and trends suggesting higher clearance values were observed in some studies, but not all, for drugs metabolized through CYP1A2.^48^ Obesity is often associated with chronic low-grade inflammation,^49^ which may decrease CYP1A2 activity. In patients with severe obesity, CYP1A2 activity increased slightly after 9 weeks of diet-induced weight loss.^50^ However, baseline CYP1A2 activity was similar in patients with severe obesity compared with a control group of normal-weight to overweight individuals.^50^ In another study, CYP1A2 activity did not differ between normal-weight, overweight, and morbidly obese patients.^51^ Further studies should be performed to better assess the influence of body weight on olanzapine plasma levels, particularly in obese patients, with better characterization of body composition (proportion of lean versus fat mass).

Our results showed that the combination with valproate significantly decreased olanzapine C:D ratios. This effect is in accordance with most previous studies that found 18%–42% lower olanzapine C:D ratios^16,18,30,52^ or 23% higher olanzapine clearance^37^ in patients taking valproate, although a few studies did not find significant effects.^19,25^ Valproate has complex effects on olanzapine pharmacokinetics, and the mechanism of this interaction remains unclear. One hypothesis is that valproate induces P-glycoprotein (P-gp) transport of olanzapine. Indeed, some studies have suggested that olanzapine could be a substrate of P-gp,^53^ and in vitro studies have shown that valproate may upregulate P-gp and CYP3A4 gene expression.^54^ A presystemic mechanism was suggested, possibly through an induction of P-gp efflux in the intestine, as olanzapine dose-adjusted concentrations decreased under valproate treatment in patients receiving oral olanzapine but not in those receiving the long-acting injection.^52^ Another hypothesis might be a displacement of olanzapine plasma protein binding by valproate. Olanzapine is highly bound to proteins, and valproate is a well-known displacer found in high concentrations in plasma.^22^ Regarding the main enzymes involved in olanzapine metabolism, valproate is not considered to be an inducer of UGT1A4 nor of CYP1A2.^52^ On the contrary, valproate is known to inhibit UGT1A4,^55^ which might increase olanzapine levels, but this was not observed in clinical studies. Valproate also inhibits UGT2B7^55^ and CYP2C9 and slightly inhibits CYP2C19 and CYP3A4^56^; however, these isoforms are not involved in olanzapine metabolism. Owing to this potential interaction, TDM might be useful for patients treated with both olanzapine and valproate with a partial antipsychotic response to optimize olanzapine dosage.

Comedication with strong CYP2D6 inhibitors (levomepromazine, fluoxetine, and/or paroxetine) or esomeprazole did not influence the olanzapine C:D ratio. CYP2D6 is a minor metabolic pathway of olanzapine,^6^ and esomeprazole is a weak CYP1A2 inducer,^31^ which may explain the absence of an observed effect. However, as the number of patients taking at least 1 CYP2D6 inhibitor (6.3%) or esomeprazole (7.8%) was small, the lack of statistical power could not be excluded. These results are in accordance with those of a previous study that observed no significant effect of CYP2D6 inhibitors on olanzapine clearance.^37^ However, in another study, comedication with paroxetine, fluoxetine, or sertraline (a weak CYP2D6 inhibitor), all 3 drugs considered as a single group of medications, increased olanzapine plasma concentrations by 32%.^25^ In another study, coadministration of fluoxetine in 15 healthy male patients was associated with a statistically significant, but clinically irrelevant, decrease in olanzapine clearance.^57^

We observed that the C:D ratios of olanzapine were significantly lower in inpatients than in outpatients. This result could be interpreted as counterintuitive because better adherence might have been expected in inpatients, in whom medication intake is frequently supervised, than in outpatients. However, the presence of severe positive symptoms, which are supposedly more frequent in inpatients, has been identified as a risk factor for nonadherence to treatment.^58^ Another hypothesis might be a selection bias with TDM performed more frequently during hospitalization in patients with poor response, which might be due to lower plasma concentrations caused by more rapid metabolism or poor adherence.

Finally, we observed an inconsistent association between CRP levels and olanzapine C:D ratios, which might be explained by the low proportion of patients with significantly elevated CRP levels in the study population. In a small study including 24 patients with pathological CRP values (>5 mg/L), a trend for a positive correlation between CRP and olanzapine C:D ratios was observed.^59^ In a larger study including 354 patients, a population pharmacokinetic model showed that the cooccurrence of infection decreased olanzapine clearance by 25% on average.^37^ Additional studies should be performed to further assess the association between CRP and olanzapine plasma concentrations.

### Limitations and Strengths

Our study has several limitations. First, due to the observational setting, we were unable to access all potential cofactors that could impact olanzapine plasma levels, such as patient adherence or dietary habits (eg, the consumption of cruciferous vegetables, which are known to induce CYP1A2 activity,^60^ could have influenced the results). The difference in olanzapine C:D ratios observed between inpatients and outpatients should be interpreted cautiously because it could be attributed to a selection bias, as explained in the Discussion section. We had a limited number of patients with significantly elevated CRP values, which impeded our ability to adequately assess the influence of inflammation on olanzapine pharmacokinetics. We did not quantify olanzapine metabolites, which could have provided valuable insights into specific metabolic pathways. However, from a clinical perspective, the quantification of olanzapine metabolites is not considered essential because they are not believed to significantly contribute to the pharmacological profile.^61^ Finally, our analysis did not include factors such as ethnicity and polymorphisms of drug-metabolizing enzymes, which could potentially influence olanzapine plasma exposure.^6^ In addition, clinical information regarding therapeutic response and adverse reactions was not incorporated into our analyses.

Nevertheless, an important strength of this study is that the most relevant intrinsic and extrinsic covariates known to influence olanzapine pharmacokinetics, such as smoking status, sex, age, body weight, and comedications, were included. Furthermore, owing to the strict predefined exclusion criteria, only adequate data were included in the analyses, such as plasma concentrations measured at a steady-state with a known sampling time after the last dose. In addition, the inclusion of repeated observations allowed us to consider intraindividual variability in various statistical models. Finally, for olanzapine plasma quantification, a precise and accurate UHPLC-MS/MS method was used in an ISO 15189 environment to improve data quality.

## CONCLUSIONS

This study investigated the impact of key intrinsic factors (such as sex, age, and body weight) and extrinsic factors (including smoking status and concomitant use of valproate) on the pharmacokinetics of olanzapine. The findings suggest that considering these factors in advance could enable the customization of olanzapine doses, potentially leading to improved therapeutic responses and reduced adverse reactions. TDM could be employed during treatment to further modify doses as needed based on clinical indications.
